# SSFLNet: A Novel Fault Diagnosis Method for Double Shield TBM Tool System

**DOI:** 10.3390/s24082631

**Published:** 2024-04-20

**Authors:** Peng Zhou, Chang Liu, Jiacan Xu, Dazhong Ma, Zinan Wang, Enguang He

**Affiliations:** 1 College of Engineering Training and Innovation, Shenyang Jianzhu University, Shenyang 110168, China; zhoupeng@sjzu.edu.cn; 2 School of Mechanical Engineering, Shenyang Jianzhu University, Shenyang 110168, China; Liuchang@stu.sjzu.edu.cn (C.L.); heenguang@sjzu.edu.cn (E.H.); 3 The College of Information Science and Engineering, Northeastern University, Shenyang 110819, China; madazhong@ise.neu.edu.cn

**Keywords:** tunnel boring machine (TBM), tooling system, hydraulic thrust system, failure localization

## Abstract

In tunnel boring projects, wear and tear in the tooling system can have significant consequences, such as decreased boring efficiency, heightened maintenance costs, and potential safety hazards. In this paper, a fault diagnosis method for TBM tooling systems based on SAV−SVDD failure location (SSFL) is proposed. The aim of this method is to detect faults caused by disk cutter wear during the boring process, which diminishes the boring efficiency and is challenging to detect during construction. This paper uses SolidWorks to create a complete three−dimensional model of the TBM hydraulic thrust system and tool system. Then, dynamic simulations are performed with Adams. This helps us understand how the load on the propulsion hydraulic cylinder changes as the TBM tunneling tool wears to different degrees during construction. The hydraulic propulsion system was modeled and simulated using AMESIM software. Utilizing the load on the hydraulic propulsion cylinder as an input signal, pressure signals from the two chambers of the hydraulic cylinder and the system’s flow signal were acquired. This enabled an in−depth exploration of the correlation between these acquired signals and the extent of the tooling system failure. Following this analysis, a collection of normal sample data and sample data representing different degrees of disk cutter abrasions was amassed for further study. Next, an SSFL network model for locating the failure area of the cutter was established. Fault sample data were used as the input, and the accuracy of the fault diagnosis model was tested. The test results show that the performance of the SSFL network model is better than that of the SAE−SVM and SVDD network models. The SSFL model achieves 90% accuracy in determining the failure area of the cutter head. The model effectively identifies the failure regions, enabling timely tool replacement to avoid decreased boring efficiency under wear conditions. The experimental findings validate the feasibility of this approach.

## 1. Introduction

A tunnel boring machine (TBM) is a piece of equipment uniting mechanical, electronic, hydraulic, and laser technologies for large−scale, industrialized tunnel excavation. It boasts advantages such as a relatively steady excavation speed, shorter construction periods, a minimized ecological impact, and overall high efficiency [1]. However, during the TBM excavation process, the harsh working environment inside the tunnel and the inherent complexity of the cutting tool system at the machine’s forefront contribute to a higher incidence of tooling system failures during construction. Among them, the wear of disk cutters accounts for 80% of TBM tool system failures, representing the largest proportion and the most important cause of TBM tool system failures. When tool wear occurs, the digging speed will decrease, resulting in a longer project cycle time [2]. In addition, tool wear leads to increased maintenance costs [3]. The frequent replacement of badly worn tools is labor−intensive and time−consuming, and also increases construction costs. Monitoring and addressing tool wear during construction poses certain difficulties. Currently, the exclusion of failure areas caused by disk cutter wear heavily relies on manual inspections during shutdowns, requiring downtime for handling and resulting in lower efficiency [4]. Therefore, tooling system fault diagnosis is a crucial task by which to address the maintenance management of TBM tooling systems.

Many researchers and scholars have extensively studied the force changes caused by different degrees of wear occurring in TBM disk cutters. Rimpault et al. [5] conducted a fractal analysis of cutting force signals, revealing that the fractal characteristics of these signals can serve as estimators for tool wear and surface quality. Ding et al. [6] proposed a micromilling tool wear prediction framework. By taking into account tool deformation, tool runout, the time−varying cutting coefficient, the chip separation state, and tool wear estimation results, a new integrated model for the real−time monitoring of the cutting force was developed. The accuracy of the proposed tool wear prediction and cutting force model was confirmed through experiments. Liu et al. [7] introduced a method for monitoring wear on a tooth−by−tooth basis in asymmetric micromilling tools. This approach was grounded in the reconstruction of chip thickness and cutting force signals. The wear status of each tooth was estimated by integrating the reconstructed cutting chip thickness and the measured cutting force signals into the proposed asymmetric micromilling force model. Wu et al. [8] carried out milling and grinding experiments utilizing electroplated diamond tools. They evaluated the advancement of tool wear by analyzing the time and frequency domains, as well as the scaled and unscaled characteristics of the cutting force and acceleration signals. Choudhury et al. [9] introduced a method for estimating tool wear in milling, which relies on the correlation between flank wear and the average tangential cutting force coefficient. This method was used to predict tool wear, and experimental verification demonstrated that the proposed model provides more accurate predictions of tool wear. Kamm et al. [10] initiated their study by analyzing the frictional interaction between the cutter tool and hard rock. They investigated the correlation between the rolling force and the propulsion force of the cutter tool, leading to the derivation of the energy equation for the cutter tool. Subsequently, they deduced the energy equation for the cutter tool used in a hard rock TBM.

In recent years, with the rapid development of information technology and the popularization and application of big data, machine learning, as a powerful tool for data analysis and recognition, has achieved remarkable results in various fields [11,12,13,14,15]. In the field of TBMs, machine learning has revolutionized traditional engineering and construction methods [16,17,18,19]. Machine learning algorithms can analyze large amounts of data intelligently, providing new insights for the operation and maintenance of TBMs. This improves the efficiency and quality of tunneling projects [20].

Machine learning introduces advanced capabilities in data analysis and prediction into TBM tool wear research. This provides robust technical support to enhance the tunnel boring machine performance, reduce maintenance costs, and improve engineering efficiency. Papenberg [21] combined a gradient−weighted class−activation mapping technique with a convolutional neural network and classified the wear of milling tools with 94.41% accuracy. Domínguez−Monferrer [22] proposed a methodology incorporating data collection, data preprocessing, linear regression, the k−nearest neighbor, and random forest ML algorithms, by which the tool wear state was identified. Ou [23] proposed a new method which involves a stacked denoising self−encoder (SDAE) combined with an online sequential extreme learning machine (OS−ELM) for the intelligent identification of tool wear states. Li [24] presented an innovative audio signal processing method. This approach utilizes a blind source separation method to distinguish the source signal from the noise and extends principal component analysis for dimensionality reduction. This enables the prediction model to effectively classify tool wear.

In the dynamics field, the researchers mentioned above have estimated tool wear. They have also proposed a model for detecting the cutting force, analyzed the evolution of tool wear, and established the energy equation for the tool. This was done through a comprehensive study of force relationships. In the realm of machine learning, scholars have proficiently identified or predicted tool wear states by integrating convolutional neural networks and utilizing machine learning algorithms like the random forest algorithm and the stacked denoising self−encoder. However, none of the above studies have integrated dynamics analysis within the field of machine learning.

**Figure 1:**
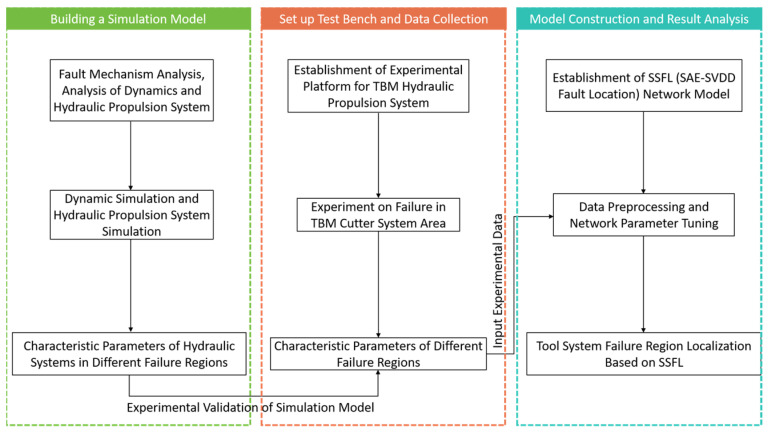
Flowchart of TBM Cutter Head Failure Localization Technique.

In response to the above issues, this paper proposes a method to localize the failure region of the tool system. The experimental process flowchart is shown in Figure 1. Firstly, this paper analyzes the failure mechanism of the disc cutter and determines the research focus of this study. Next, three−dimensional modeling of the tool system and the hydraulic main propulsion system is conducted. The dynamics of the hydraulic propulsion system are simulated and analyzed using Adams software (version 2020). Finally, the paper constructs a tool system fault diagnosis method based on SSFL.

## 2. Disk Cutter Failure Mechanism Analysis

**Figure 2:**
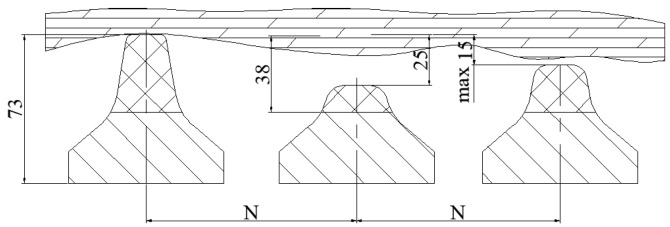
Position map of disk cutter wear tool.

During TBM tunneling construction, the hydraulic propulsion system generates thrust, which is then transmitted through the cutter plate to penetrate the rock and break it. The cutter plate performs circumferential movement with its palm face center as the pivot. In the process of rotary rock breaking, the wear type and degree of the disk cutter are influenced by various factors, resulting in varying levels of change. Based on the characteristics of the wear pattern, it can be classified into two types: the normal wear and abnormal wear of the disk cutter. Normal wear arises from rolling friction between the cutter ring and the palm surface. Over the operational time, the cutter ring’s diameter diminishes while the width of the cutter edge steadily increases. When the cutter edge width surpasses the rated value, it is considered the standard for disk cutter wear failure, as depicted in Figure 2. According to the actual engineering data statistics, the disk cutter in the rock−breaking process of the cutter circle undergoes normal wear of more than 80% of the total wear. Therefore, in the process of digging, the uniform wear of the disk cutter is one of the main influencing factors of the failure of the TBM tool system, and it is especially important to diagnose the degree of disk cutter wear.

The abnormal wear of the disk cutter refers to uneven wear caused by various factors, resulting in different types of wear such as biased grinding, chipping, displacement, or the dislodgement of the cutter ring, as well as bearing damage. These issues are primarily due to external factors, leading to distinct forms of failure. The probability of failure due to abnormal wear is extremely low compared to normal wear. Therefore, this paper focuses on addressing disc cutter failures caused by normal wear.

## 3. Dynamics Simulation Analysis

### 3.1. Disk Cutter Arrangement

**Figure 3:**
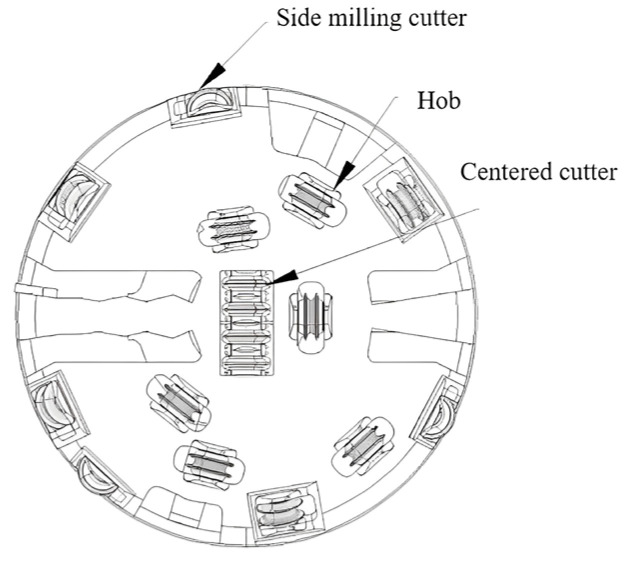
Disk cutter layout.

In this study, a double−shield TBM with a cutter diameter of 5500 mm is used as an engineering example for simulation analysis. Figure 3 illustrates the arrangement of the disk cutter, encompassing the center disk cutter, side disk cutter, and positive disk cutter. The disk cutter experiences wear of 5 mm and 15 mm simultaneously. By comprehensively considering the constraints within the system and the contact mode, a dynamics model of the drive system is established using Adams software for simulation analysis, as depicted in Figure 3.

### 3.2. Main Thrust Hydraulic Cylinder Arrangement

**Figure 4:**
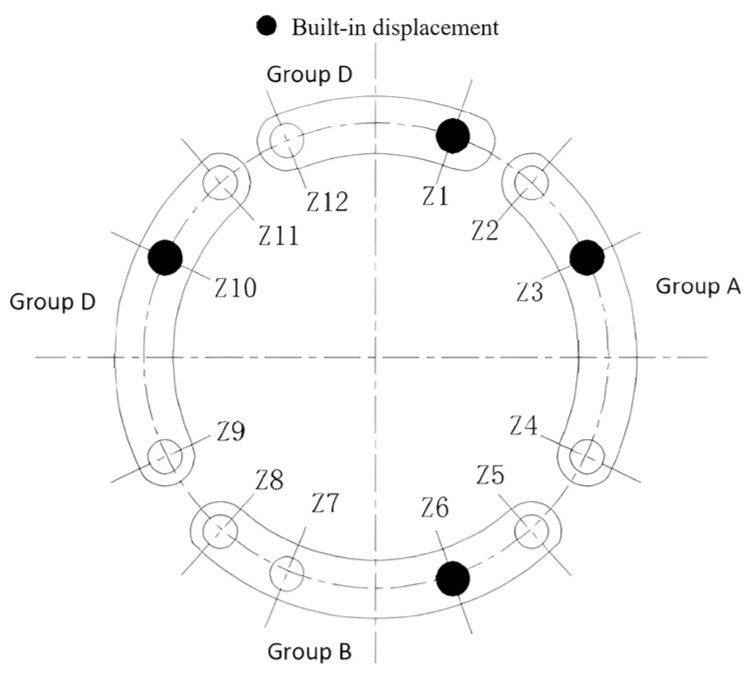
Main thrust hydraulic cylinder layout.

The arrangement of the hydraulic cylinders is divided into four regions. In the actual project, to minimize hydraulic system costs and consider TBM directional control, the primary thrust cylinders are grouped into four regions for coordinated control. Each region includes a cylinder equipped with a displacement sensor, as depicted in Figure 4, illustrating the zoning arrangement of the main push cylinders. The hydraulic cylinder monitored in this dynamics simulation is Z3 of group A. Finally, the load variation curve of the propulsion hydraulic cylinder is obtained when the disk cutter undergoes different degrees of wear.

### 3.3. Adams Dynamics Simulation

**Figure 5:**
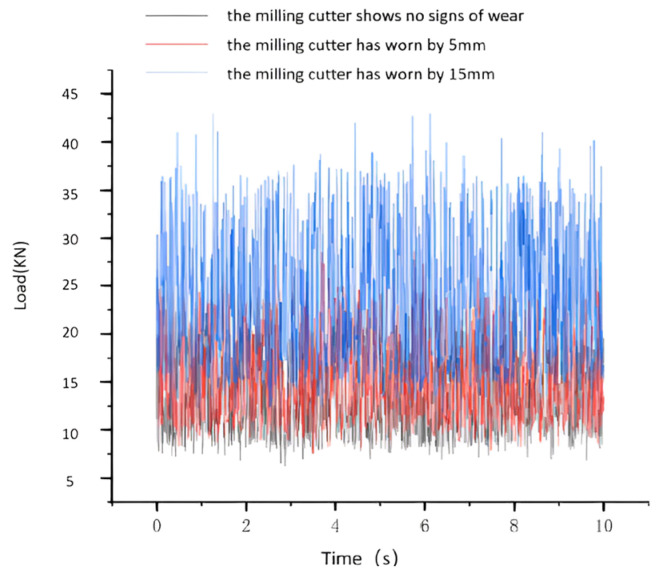
Load curve of a single disk cutter.

**Figure 6:**
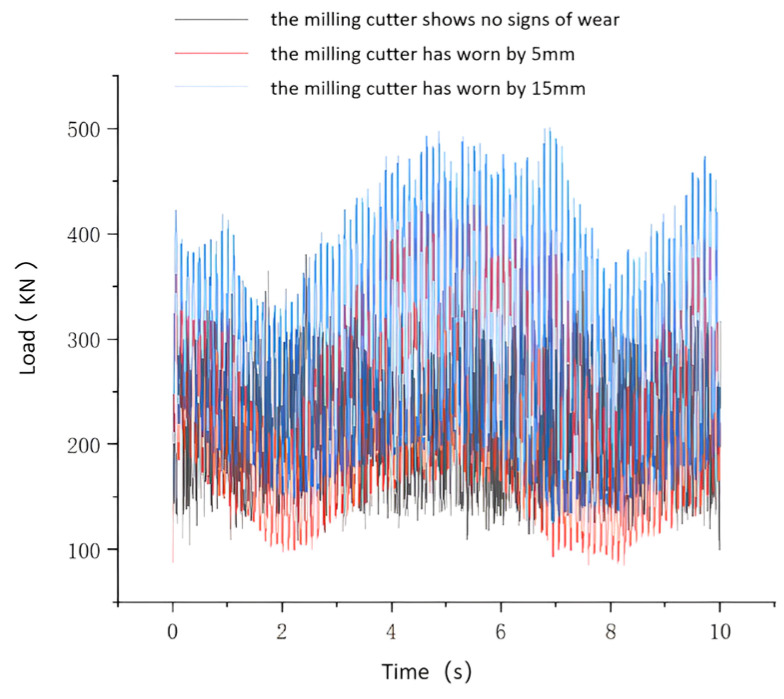
Load curve for hydraulic cylinders.

It is assumed that the cutting force undergoes periodic changes and the rock being cut is a single type of marble, rather than a composite rock formation. The process of TBM disc cutters breaking rock is simulated using this method. The simulated results are shown in Figure 5 and Figure 6. In Figure 5, load curves are depicted for different degrees of wear when there is no failure area on the cutter. The only difference between Figure 5 and Figure 6 is that Figure 6 represents a failure occurring on the cutter. The TBM cutter system and hydraulic main propulsion system were modeled in SolidWorks for three−dimensional representation. The dynamic simulation analysis of the hydraulic propulsion system was conducted using Adams software. Based on experimental data, load curves for the cutter at different degrees of wear were derived. The loads under normal breaking work conditions (i.e., 5 mm and 15 mm wear) were applied to each cutter. The simulation results illustrated the variation of loads on the propulsion hydraulic cylinder under normal conditions and two different degrees of wear, as well as forward excavation, as shown in Figure 5 and Figure 6. Figure 5 and Figure 6 represents the load variation on the propulsion hydraulic cylinder when the cutter is not worn and worn, respectively. The x−axis represents the operation time of the propulsion hydraulic cylinder, and the y−axis represents the load variation of the hydraulic cylinder. As shown in Figure 5 and Figure 6, when the cutter is not worn, the hydraulic cylinder exhibits a relatively smooth load curve with stable fluctuations around a certain value. However, when the cutter is worn, the load fluctuations of the hydraulic cylinder increase. Additionally, the load curve is smoother compared to the condition of the cutter head failing. These results provide data support for the simulation analysis of the TBM hydraulic propulsion system.

**Figure 7:**
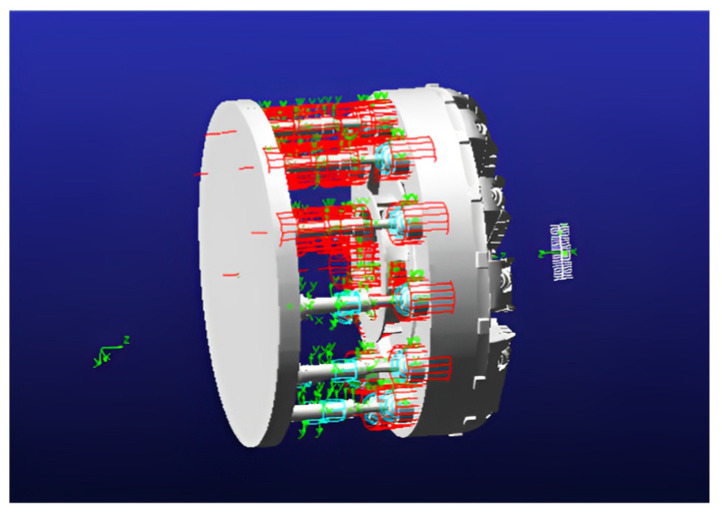
Hydraulic main propulsion system model.

The hydraulic cylinders are configured as a highly rigid spring system. Both the propulsion hydraulic cylinders and the cutter are constrained using cylindrical sub−restraints. Using a dynamic simulation, this paper establishes the rotational speed of the cutter, the vertical force on the disk cutter, the propulsion speed, and the force exerted on the propulsion hydraulic cylinder under normal disk cutter conditions and at different degrees of wear. The simulation process is illustrated in Figure 7.

## 4. AMESIM Hydraulic Simulation Analysis

### 4.1. Principle of the Hydraulic System

**Figure 8:**
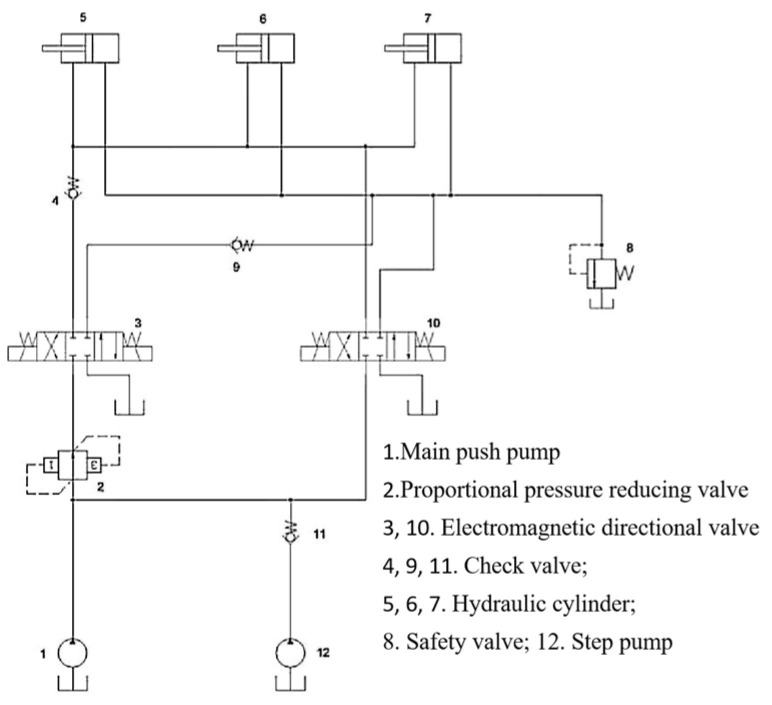
Hydraulic schematic diagram of TBM main thrust system.

The design of the main thrust hydraulic system requires that each group of cylinders can achieve coordinated action. Each group can have independent pressure regulation capabilities, enabling step changes and ensuring the rapid action of the main thrust cylinder. Each control principle for the group of cylinders is the same. Figure 8 shows the main push cylinder hydraulic schematic diagram. Among them: 1. Main push pump; 2. Proportional pressure−reducing valve; 3, 10. Electromagnetic directional valve; 4, 9, 11. Check valve; 5, 6, 7. Hydraulic cylinder; 8. Safety valve 12. Change pump.

When the main thrust cylinder is used for propulsion, energizing the left position of electromagnetic reversing valve 3 allows high−pressure oil to enter the rodless cavity of the hydraulic cylinder through proportional pressure−reducing valve 2 and check valve 9 of electromagnetic reversing valve 3. When the main push cylinder needs to be quickly extended, main push pump 1 and step−changing pump 12 provide a large flow rate of the oil source, the right position of electromagnetic reversing valve 10 is energized, and the main push cylinder is quickly extended.

### 4.2. Modeling of Hydraulic Cylinders

**Figure 9:**
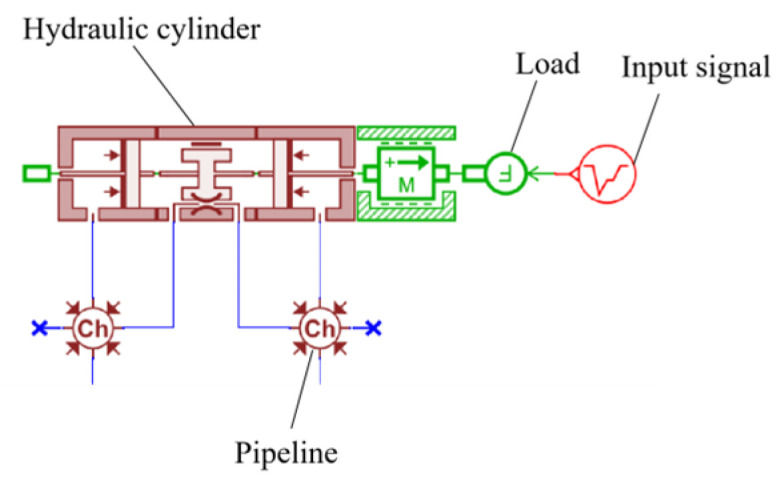
Hydraulic cylinder AMESIM model.

As the TBM adjusts positions by altering the speed of each hydraulic cylinder during the excavation process, the hydraulic cylinder model must consider variations in the chamber volume and the effects of hydraulic cylinder leakage. Therefore, the flow within the hydraulic system must maintain continuity to ensure the hydraulic cylinder forces meet the equilibrium requirements. The flow continuity equation of the hydraulic cylinder is as follows:

(1) $$nD_{n}=\lambda p+\frac{v\left(y\right)}{2\beta_{e}}\cdot \frac{d_{p}}{dt}+A\frac{dy}{dt}$$

where n denotes the rotational speed of the hydraulic pump;${\;d}_{p}$ denotes hydraulic pump displacement; λ indicates the hydraulic cylinder leakage factor; p represents the working pressure of the rodless chamber of the hydraulic cylinder; $v\left(y\right)$ represents the volume of the cavity without a piston rod end; $\beta_{e}$ represents the effective volumetric modulus of the elasticity of the fluid; A represents the cross−sectional area with rod ends; and y represents the motion displacement of the piston rod. The force balance equation for a hydraulic cylinder is:

(2) $$F=Ap_{1}=m\frac{dv}{dt}+Bv+Kx+F_{load}$$

where F represents the propulsive force of the hydraulic cylinder; A represents the area of the rodless end of the hydraulic cylinder; $p_{1}$ indicates the working pressure of the hydraulic cylinder; m denotes the load mass; v indicates the speed of the piston rod of the hydraulic cylinder; B denotes the damping factor of the system; K denotes the hydraulic cylinder stiffness; x denotes the displacement of the piston rod of the hydraulic cylinder; and $F_{load}$ indicates the load of the hydraulic cylinder. The required hydraulic cylinder submodule is selected from AMESIM’s component library to establish the simulation model, as shown in Figure 9.

Based on AMESIM’s HCD library, the composite requirements of the hydraulic cylinder submodule were selected, and its parameters were set accordingly. The basic parameters of the hydraulic cylinder are shown in Table 1.

### 4.3. Modeling of Hydraulic Pumps

**Figure 10:**
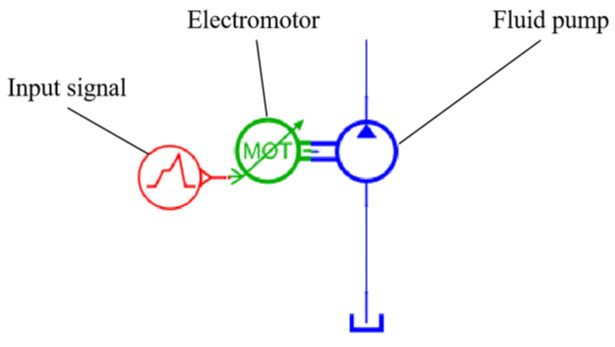
Hydraulic pump AMESIM model.

The hydraulic pump chosen for the propulsion hydraulic control system designed in this study is an axial piston pump. Modeling the axial piston pump in AMESIM primarily involves establishing the sliding shoe vane and the oil distribution plate. Due to its intricate structure, the modeling process for the hydraulic pump directly utilizes the basic model available in the hydraulic component library, as illustrated in Figure 10.

The output of the hydraulic oil in the direct−drive pump control system is primarily controlled by the speed of the hydraulic pump. Therefore, the key factor involves setting the displacement of the hydraulic pump using the basic parameters of the hydraulic pump, as shown in Table 2.

### 4.4. Modeling of Open−Loop Simulation of Hydraulic Propulsion System

**Figure 11:**
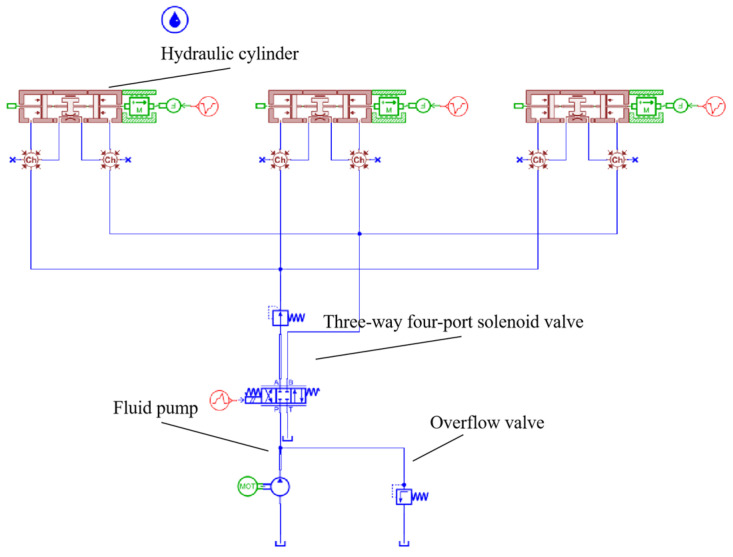
AMESIM Simulation.

Based on the working principles of the shield tunneling machine, the hydraulic propulsion system was simplified. Using the previously established hydraulic system component models, schematics were recreated within AMESIM software (version 2021.1). Following the schematic in Figure 8, an open−loop simulation system model was constructed, resulting in the creation of the single−loop open−loop simulation model for the hydraulic propulsion system depicted in Figure 11.

### 4.5. Hydraulic Propulsion System Open−Loop Simulation Results

**Figure 12:**
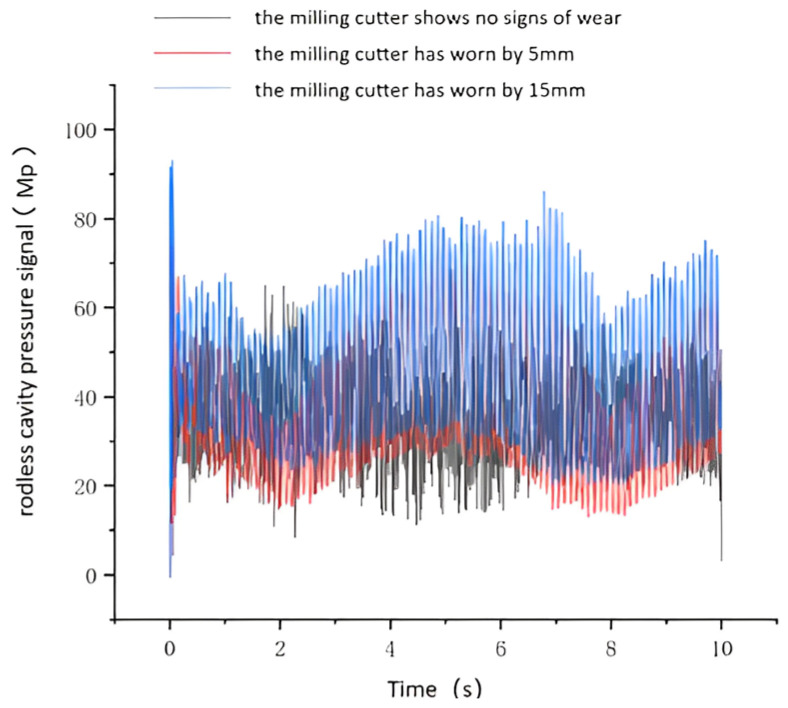
Rodless chamber pressure in normal and faulty condition.

**Figure 13:**
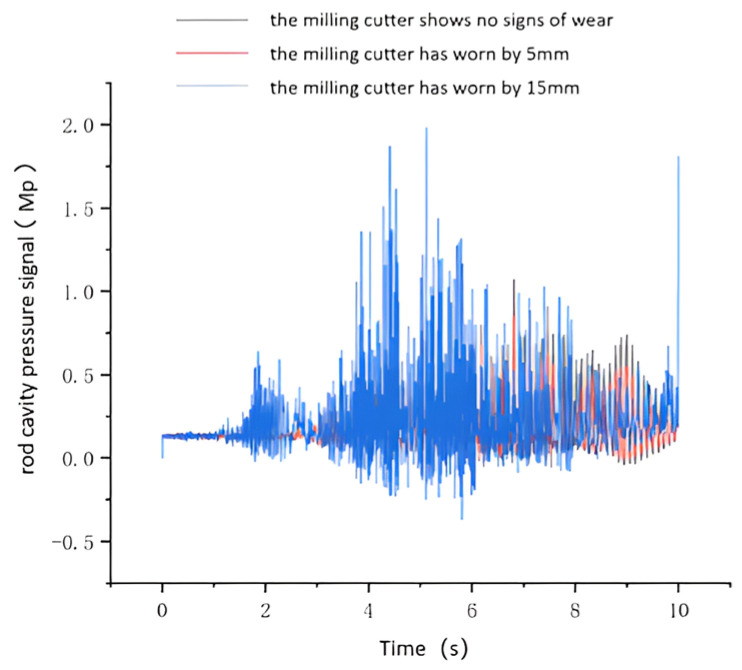
Rod chamber pressure signal in normal and fault condition.

The load variation data obtained from the kinetic simulation of the hydraulic cylinder were input into the hydraulic simulation system. The internal parameters for the hydraulic cylinder, hydraulic pump, and other hydraulic components were configured for simulation purposes to derive the results. Figure 12 and Figure 13 display the pressure signals obtained from the rodless and rodded chambers of the hydraulic cylinder under normal conditions, as well as the pressure signal from the rodless chamber of the hydraulic cylinder under fault conditions. The figure illustrates that under unworn disk cutter conditions, the pressure signal in the rodless cavity remains stable. However, when the disk cutter wears, the rodless cavity’s pressure signal fluctuates significantly. This pressure signal variation in the rodless cavity serves as valuable data support for diagnosing tooling system failures.

## 5. SAV−SVDD Failure Location Method (SSFLNet)

In this section, the SSFLNet is introduced as a new method for identifying tool system failure regions. The development of this solution was prompted by the intricate nature of the TBM tool system, the proximity of the disk cutter arrangement, the challenging monitoring of disk cutter signals, and the irregularities observed in the time−domain pressure signals from the rear hydraulic propulsion system.

### 5.1. SAE Feature Extraction Module

A Stacked Autoencoder (SAE) is a deep neural network composed of multiple Autoencoders (AE) stacked together. In this section, the paper utilizes the pressure signals from various regions during the cutter’s operation as multi−channel input data $X\in R^{C\times T}$ to establish the feature correlation between cutter vibration signals and the failure region. Here, C represents the number of channels for the pressure signals, while T denotes the number of sampling points in these signals. Then, the multi−layer self−encoder is used to extract the failure features.

For the input pressure signal, in order to map it to the feature space, the parameter mapping function ${X\to f}_{W_{i},\theta_{i}}(X)$ must be established, where $W_{i}=\{W_{ei},\hat{W_{ei}}\},\;\theta_{i}=\{\theta_{ei},\hat{\theta_{ei}}\}$.

In order to make the output of the function infinitely close to the input X:

(3) $${\hat{X}=f}_{W_{i},\theta_{i}}(X)\approx X$$

The hidden layer feature node can be represented as:

(4) $$Y_{i}=g(X)=\phi_{i}(XW_{ei}+\theta_{ei})$$

(5) $$\phi_{i}(t)=\frac{1}{1+e^{-t}}$$

As a result, the reconstructed vector of input X can be obtained by inverse mapping:

(6) $$\hat{X}=g(X)=\phi_{i}(Y_{i}\hat{W_{ei}}+\hat{\theta_{ei}})$$

Finally, the reconstruction error is obtained:

(7) $$J_{E}(W_{i},\theta_{i})=\frac{1}{Num}\sum_{t=1}^{Num}\frac{1}{2}{\left\|{\hat{X}}_{t}-X_{t}\right\|}^{2}$$

### 5.2. SVDD Feature Classification Module

**Figure 14:**
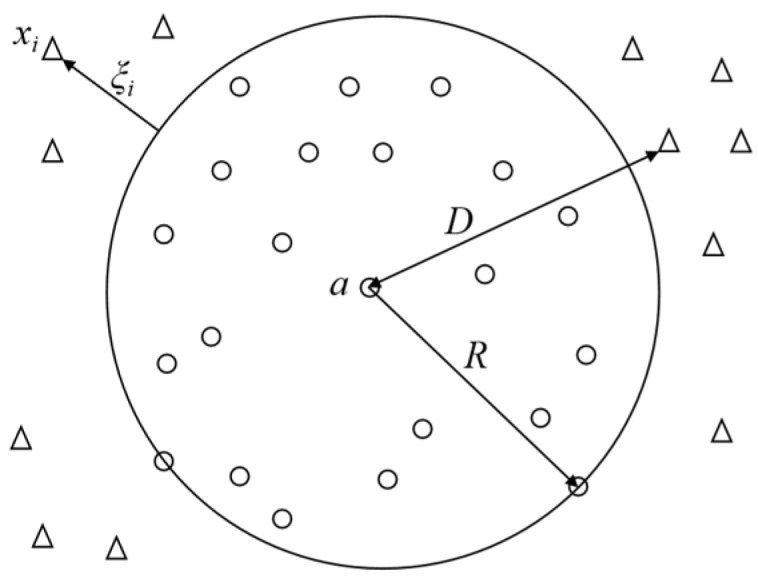
Schematic of SVDD hypersphere classification.

For the division of blade failure regions, the smallest volume hypersphere is employed to differentiate between the operational and failure states of each region. This method is called SVDD (Support Vector Data Description). This is achieved by defining a center point and a radius to classify the state characteristics, as depicted in Figure 14.

This hypersphere serves as a decision boundary, distinguishing positive class samples from negative class samples. It identifies the feature classes of test sample points based on their distance relationship with the hypersphere. To obtain this hypersphere, the constraints of this sphere are obtained according to the support vector machine theory.

(8) $${∥x_{i}-a∥}^{2}\leq R^{2},i=1,2,\cdots ,N$$

Then, the minimal hypersphere can be denoted as:

(9) $$min\left[f\left(R,a,\xi \right)\right]=min\left[R^{2}+C\sum_{i=1}^{N}\xi_{i}\right]$$

where C is the penalty parameter.

To avoid the influence of anomalous samples within the target samples on the hypersphere, a relaxation factor ξ is introduced. It allows some samples to exist outside the hypersphere, thereby enhancing the robustness of the algorithm.

(10) $$minL=R^{2}-\sum_{i=1}^{n}a_{i}\left(R^{2}+\xi_{i}-{∥\varphi \left(x_{i}\right)-a∥}^{2}\right)-\sum_{i=1}^{n}\xi_{i}\mu_{i}+C\sum_{i=1}^{n}\xi_{i}$$

The distance from the test sample to the hypersphere can be expressed as follows:

(11) $$D_{min}=∥z-a∥=z^{2}-2\sum_{i=1}^{N}\alpha_{i}\left(z\cdot x_{i}\right)+\sum_{i=1}^{N}\sum_{j=1}^{N}\alpha_{i}\alpha_{j}\left(x_{i}\cdot x_{j}\right)$$

### 5.3. Training of Network Models

**Figure 15:**
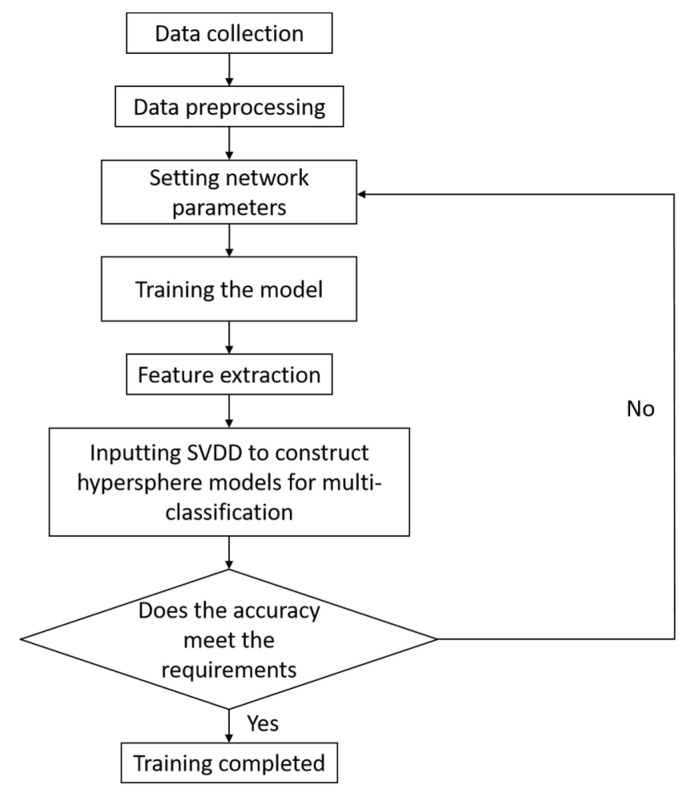
SSFL Algorithm Flowchart.

The flowchart for diagnosing the failure area of the TBM cutterhead based on the SSFL network is shown in Figure 15. The primary factors influencing SSFLNet’s performance include the number of layers in network training, neural unit count per hidden layer, epoch value, batch size, and learning rate. Whether the settings of these hyperparameters are reasonable or not is closely related to the training speed of the SSFL network and the accuracy of the classification results. In this paper, these settings were systematically adjusted based on the network’s training speed and classification accuracy to discover the optimal hyperparameters. The aim of this approach was to expedite training while upholding high accuracy levels.

Given that the number of network layers significantly affects both network results and performance, two layers were used as the initial value for the SSFLNet’s network layer hyperparameter. Subsequently, the number of network layers was incrementally increased by one. The number of neural units in each hidden layer was determined based on the equal variance series. The batch−size value was set to 500, the epoch value was set to 500, and the learning rate was set to 0.001. The experimental data were partitioned into an 8:2 ratio, with 8 portions used for training data and 2 portions reserved as the test set. The influence of each hyperparameter on the performance of SSFLNet was investigated using the control variable approach.

**Figure 16:**
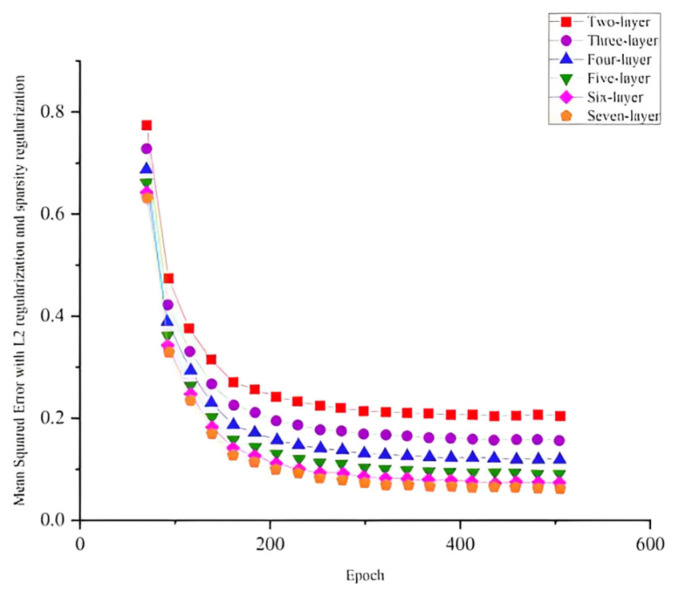
Training error plots for networks with different number of layers.

The training mean square loss for these networks is depicted in Figure 16. In Figure 16, it is evident that within the range of 2 to 5 network layers, as the depth of the network layers increases, the error value decreases more rapidly and converges to a smaller value at the final stage. When the number of network layers set up was increased from two to seven, the final test accuracy increased from 76% to 95.3%. Nevertheless, upon reaching 5 or more network layers, further increases did not notably enhance either the rate of error reduction or the test accuracy (which consistently remained around 95%). This indicates that, for our blade dataset, having 5 network layers is sufficient to capture adequate feature information. In addition, when the number of network layers increased from 2 to 7, the training time of the network increased from 33 s to 67 s. An excessively deep network tends to unnecessarily prolong the training time without significant performance gains. Consequently, the optimal network performance was achieved when utilizing 5 network layers. Specifically, the number of neural units in each layer was set to 200, 150, 100, 80, 60, and 50.

## 6. Experimental Results and Comparative Analysis

### 6.1. Data Description

**Figure 17:**
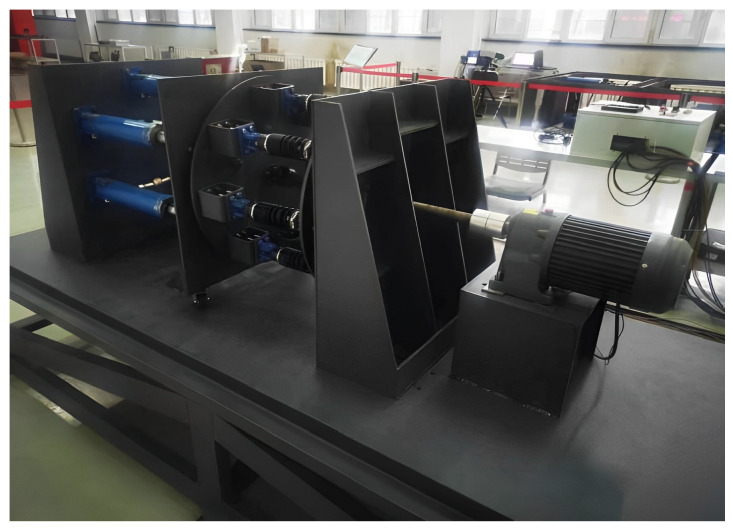
Actual lab bench construction.

Tool system failure experiments are conducted on the experiment platform of the TBM’s hydraulic propulsion system, as shown in Figure 17. The propulsion hydraulic cylinder adjusts the radial force on the propulsion plane by changing the initial linear length of adjustable springs, thus simulating failures in different areas of the tool system. The experiment divides the load simulation system into four areas (upper, lower, left, and right zones).

The experiment data are from five different categories, as shown in Table 3. The hydraulic system operating pressure is adjusted to 6–7 Mpa and the speed is set at 2 r/min. A total of 3000 feature data points are collected. Among these, 2700 data points are used for training and 300 data points are for testing.

### 6.2. Analysis of the Results

In the simulation experiments on tool system failure, this experiment collected data on cutter plate pressure during regular tool system operation and across four distinct failure categories in different regions. A total of 500 sample sets were collected for each category, amounting to a total of 1500 sample sets. The five classes of sample data were split into training and testing sets at a ratio of 8:2. Ablation experiments were conducted to validate the performance of the developed SSFLNet network. This process involves identifying operational data from the tested tool systems and evaluating the accuracy of their failure regions, as well as the training time, as detailed in Table 4. It illustrates a notable positioning error when using SVDD directly to identify the failure region of the tool system. SAE is applied to extract disk cutter failure features and subsequently employ SVM (Support Vector Machine). This can identify the failure region, which demonstrates considerable enhancements compared to directly determining the failure data. However, our proposed SSFLNet algorithm achieves even higher accuracy in identifying the failure region of the tool system. In terms of training time, SVDD trains faster, but has lower accuracy on the test set. SAE−SVM and SSFL, however, achieve higher accuracy, with SSFL requiring significantly less training time compared to SAE−SVM.

**Figure 18:**
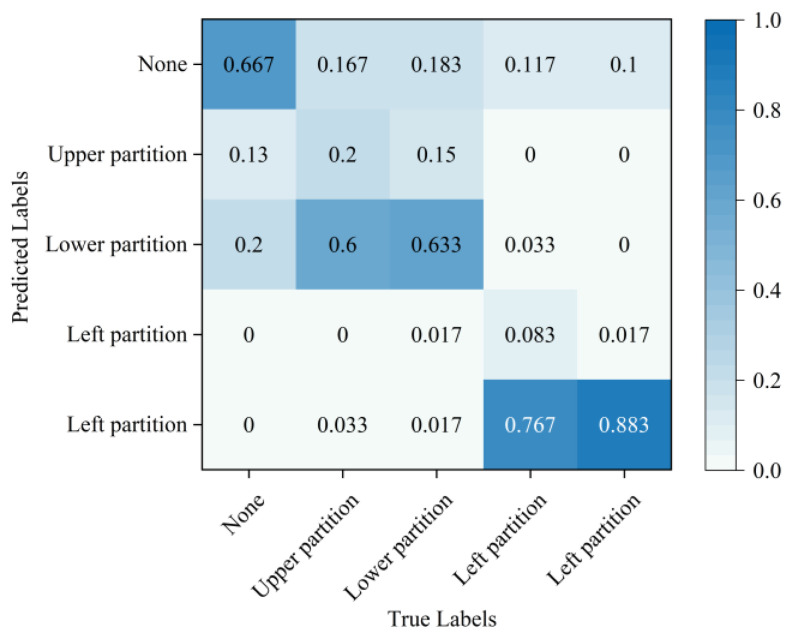
SVDD model confusion matrix.

**Figure 19:**
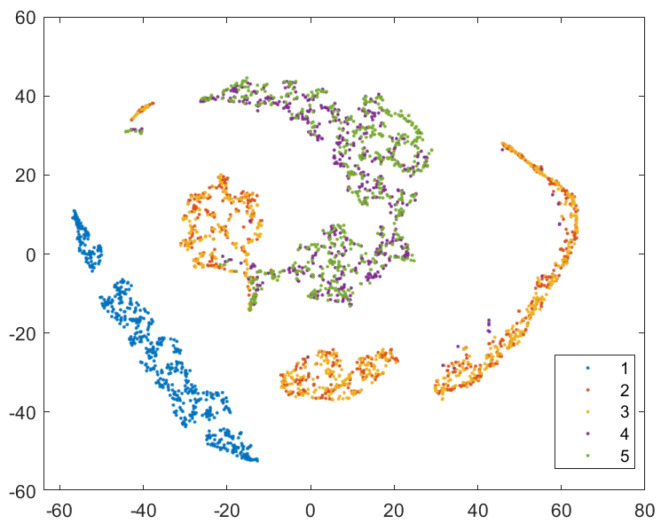
Visualization of SVDD model classification results.

**Figure 20:**
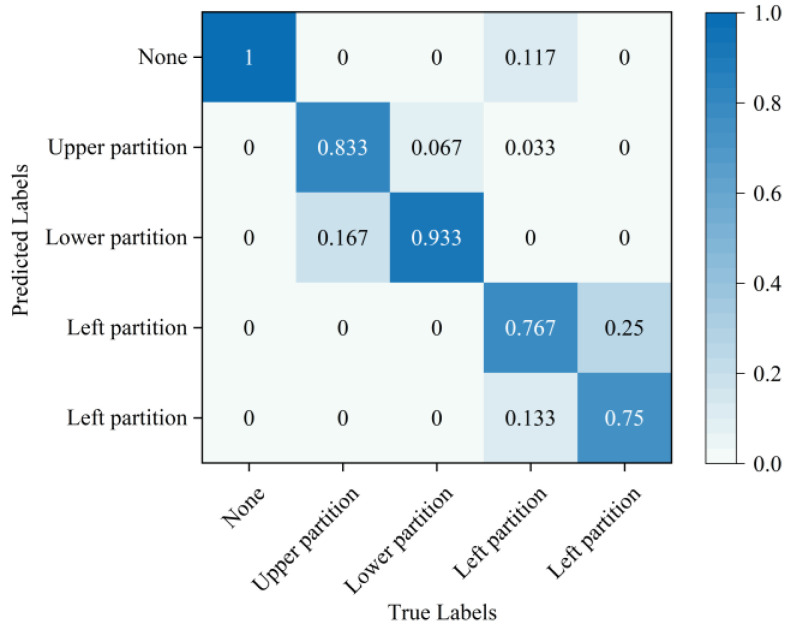
SAE−SVM model confusion matrix.

**Figure 21:**
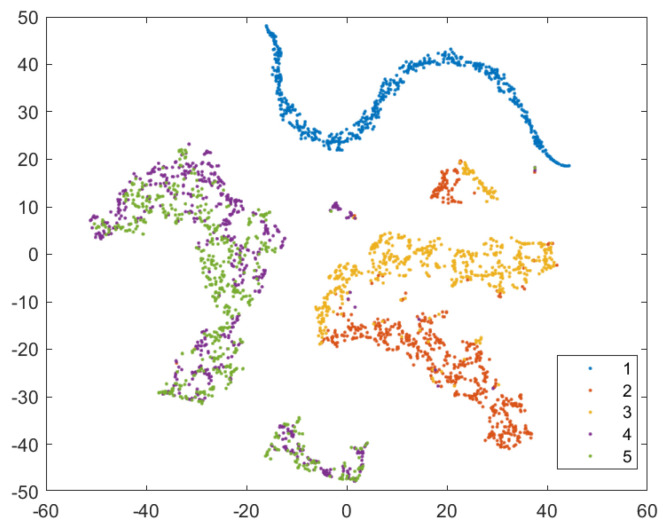
Visualization of SAE−SVM model classification results.

**Figure 22:**
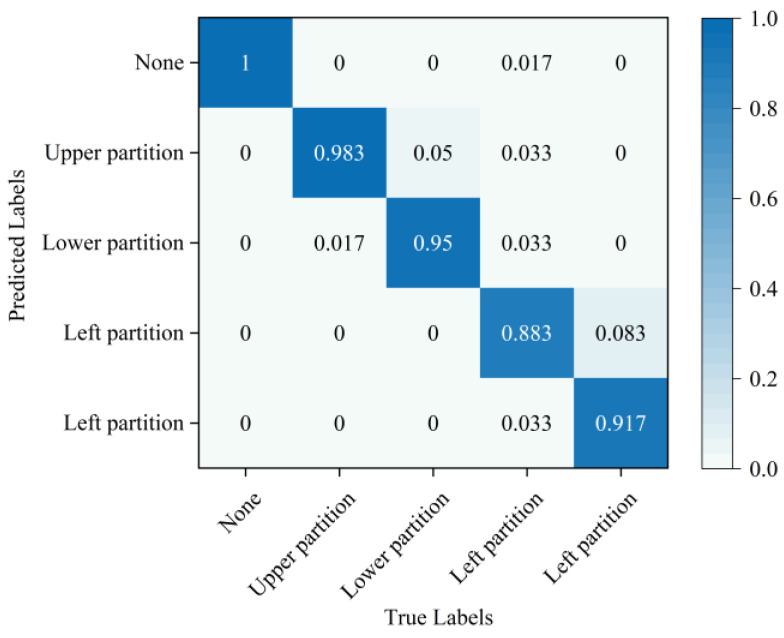
SSFL model confusion matrix.

**Figure 23:**
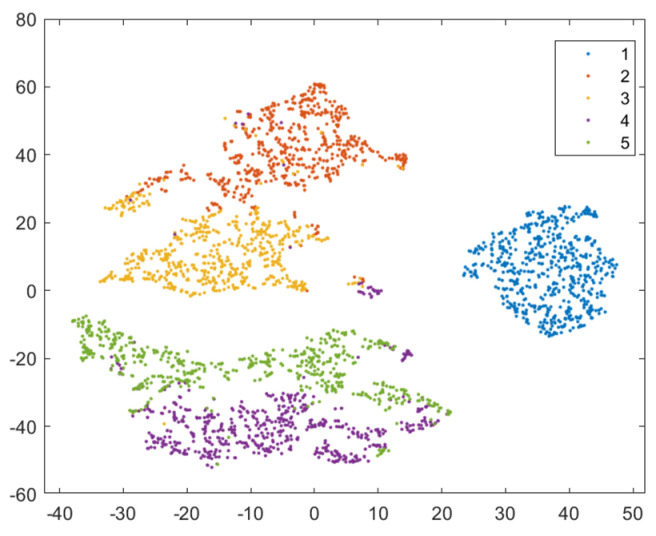
Visualization of SSFL network classification results.

In order to provide a more intuitive analysis of the performance of the three methods in Table 3 for determining the blade failure region, this paper visualized the features of the SVDD, SAE−SVM, and SSFLNet methods proposed and analyzed their confusion matrices and visualizations, respectively. In which, Figure 18 and Figure 19 respectively are the confusion matrix and visualization results of SVDD; Figure 20 and Figure 21 respectively are the confusion matrix and visualization results of SAE−SVM; Figure 22 and Figure 23 respectively are the confusion matrix and visualization results of SSFL. In Figure 19, label 1 (blue dots) denotes the data samples where no failure occurred in the TBM tool system. Labels 2 to 5 represent failures in distinct regions: upper partition failure, lower partition failure, left partition failure, and right partition failure, respectively. Figure 18 presents the outcomes of categorizing various operational data for direct tool system failure region determination using the traditional SVDD algorithm. It is evident that the accuracy of feature determination for regions 2 and 4 failures is low. In Figure 23, the failure features from regions 2 and 3 are clustered together, while features from failure types 4 and 5 overlap. When employing the SSFL method, as depicted in Figure 23, categories 2 and 3 exhibit distinct separation in their features, with only minimal overlapping feature points observed in categories 4 and 5. This corresponds to Figure 22, leading to significantly improved accuracy for each category in Figure 22.

Finally, SSFLNet was utilized to determine the failure regions of the test data, with the confusion matrix shown in Figure 22 and the classification results visualized in Figure 23. In the feature visualization graph presented in Figure 23, SSFLNet demonstrates a superior ability compared to SVDD and SAE−SVM in effectively distinguishing between the five categories of blade runtime data. This distinction results in the dispersion of feature points from different categories into separate feature clusters, minimizing any overlap between the clusters. This notable separation largely mitigates the issue of the feature cluster overlap observed in Figure 21 and Figure 23. The data in category 1 within the figure are densely distributed and exhibit a substantial separation from feature data belonging to other categories. Moreover, compared to Figure 20, when the tool system remains unaffected (category 1), SSFLNet achieves a feature classification accuracy of 100%. This performance showcases SSFLNet’s capability to effectively distinguish between normal tool system conditions and instances of tool system failure. The failure features from various partitioned tool systems exhibit band−like arrangements. Despite the close proximity of clusters representing two adjacent feature types, they are effectively differentiated with an accuracy of over 90%, as depicted in Figure 23.

The SSFLNet network proposed in this study aligns with the practical needs of TBM tool system failure diagnostics by accurately pinpointing failure areas within collected cutter area data. This precision contributes to ensuring the reliability and safety of TBM tool system operations.

**Figure 24:**
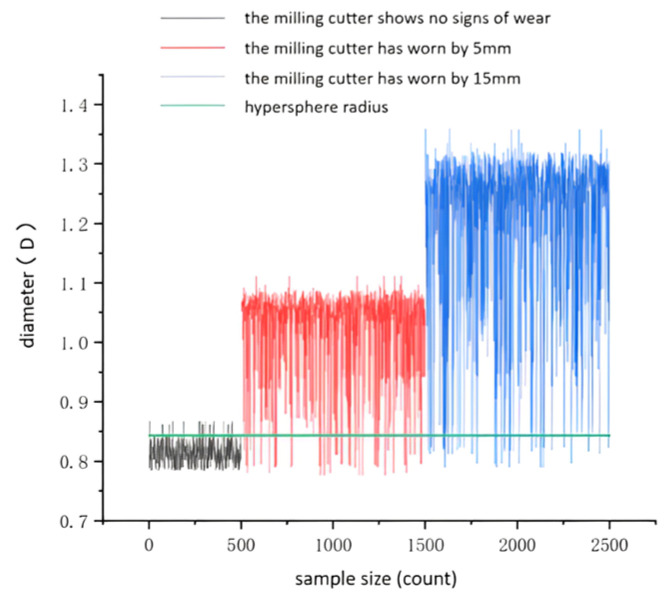
Maximum and minimum constructed model test results.

Hence, the selection of minimum and maximum eigenvalues enabled the formation of a sample set for constructing the SSFL disk cutter wear fault diagnosis model of the tool system. This fault diagnosis model was then compared against the mean, median, and mean square methods. The specific process involved setting the Gaussian kernel function parameter as σ = 1. Using this parameter, two sets of eigenvalues—composed of the maximum and minimum values corresponding to the two−cavity pressure—were formed. Subsequently, these sets were utilized to individually construct the wear fault diagnosis model of the TBM tool system. The model test results are shown in Figure 24.

From the graph, it is evident that the SSFL fault diagnosis model, constructed using the sample set comprising minimum and maximum values extracted from the rodless cavity pressure of the hydraulic cylinder, achieves an accuracy of 91.98% in diagnosing wear within the TBM tooling system. This accuracy surpasses the results obtained from the fault diagnosis model constructed using other eigenvalue combinations. Consequently, it satisfactorily meets the demand for diagnosing wear in the TBM disk cutter.

## 7. Conclusions

This study introduces the TBM tool system and proposes the use of the SSFL method to address tool system fault areas. This method enables one to monitor the main hydraulic propulsion system of the TBM during its construction, facilitating the diagnosis of tool system faults. Subsequently, the arrangement of the cutter plate and main hydraulic cylinder was simulated, and dynamic simulations were conducted using Adams software to obtain load data for individual hydraulic cylinders. Further hydraulic simulation analysis was conducted using AMESIM to analyze the pressure signals of the rodless and rodded cavities of the hydraulic cylinders. Lastly, the training error, testing accuracy, and testing time of networks with varying numbers of layers are aimed to be evaluated. The experimental results will be compared with other network models proposed in this paper (utilizing SVDD and SAE−SVM). This study aims to validate the superiority of SSFL through a confusion matrix and T−SNE visualization. From the classification results of the network, it can be observed that the classification accuracy of the tool system without failure is as high as 100%, and the classification accuracy of failure in different zones of the tool system is greater than 90%, meeting the requirements for discriminating the failure area of the dual−shield TBM tool system.

The mechanical analysis of the cutter plate serves a dual purpose. It not only offers genuine and rational data support for the algorithm, but also establishes a model enabling the diagnosis of disk cutter wear during the TBM construction and boring processes. The SSFL method’s advantage lies in its independence from the Gaussian distribution within the data, making the model highly adaptable and adept at handling outliers. This flexibility allows for a more comprehensive modeling approach, effectively capturing data characteristics and structure. As a result, the fault diagnosis model has high accuracy, ensuring feasibility and effectiveness in fault diagnosis. The utilization of the SSFL−based method enabled us to precisely pinpoint the tool failure area. This not only enhanced productivity and equipment safety, but also introduced novel solutions for analogous issues in the future. The method represents a significant innovation, contributing to advancements within the industrial domain.

## 8. Patents

Invention Name: Simulation Experimental Equipment for Double−Shield TBM Hydraulic Propulsion System.

Patent Holder: Shenyang Jianzhu University.

## Figures and Tables

**Table 1:** Characteristic parameters of the hydraulic cylinder model.

Parameter Name	Parameter Value
Hydraulic cylinder bore	280 mm
Hydraulic cylinder piston rod diameter	220 mm
Hydraulic cylinder stroke	1650 mm
Cross−sectional area of rodless piston end cavity	$3.8\times 10^{-2}\;m^{2}$
Rodless piston end chamber volume	$1.9\times 10^{-2}\;m^{3}$
Cavity cross−sectional area of rod piston end	$1.8\times 10^{-2}\;m^{2}$
Rodded piston end chamber volume	$0.9\times 10^{-2}\;m^{3}$
Leakage factor for hydraulic cylinders	$7\times 10^{-11}\;\frac{m^{3}}{s}P$
Damping factor of the system	$1\times 10^{-6}\;N\cdot m/s$
Modulus of elasticity of hydraulic fluid	$1\times 10^{8}\;N/m$

**Table 2:** Characteristic parameters of the hydraulic pump model.

Parameter Name	Parameter Value
Hydraulic pump displacement	$1.38\;\times \;10^{-5}$ L/min
Maximum speed of hydraulic pump	1450 r/min
Hydraulic pump working pressure	35 MP

**Table 3:** Experiment classification.

Tooling System Failure Zone	Adjusting the Spring	Operating Pressure in MPa	Rotation Speed (r/min)	Number of Experiments (Loops)
None	No adjustment made	0.64	1.9	610
Upper partition	Upper side	0.68	1.8	610
Lower partition	Lower side	0.65	1.9	680
Left partition	Left side	0.67	2	680
Right partition	Right side	0.65	2	620

**Table 4:** Model results comparison.

Tooling System Failure Zone	SVDD Model Accuracy	SAE−SVM Model Accuracy	SSFL Model Accuracy
None	66.7%	100%	100%
Upper partition	20%	83.3%	98.3%
Lower partition	63.3%	93.3%	95%
Left partition	8.3%	76.7%	88.3%
Right partition	88.3%	75%	91.7%
training time	203 s	459 s	323 s

## Data Availability

Data are contained within the article.
